# The complete mitochondrial genome of leafhopper *Atkinsoniella nigrita* (Hemiptera: Cicadellidae) with the shortest *12S* rRNA and longest *tRNA-Lys* of the *Atkinsoniella* genus

**DOI:** 10.1080/23802359.2023.2228932

**Published:** 2023-06-29

**Authors:** Hu Li, Kai Yu, Rui Zhao, Gang Wu, Chuan-Feng Xiong

**Affiliations:** Shaanxi Key Laboratory of Bio-resources; School of Biological Science & Engineering, Shaanxi University of Technology; Qinling-Bashan Mountains Bioresources Comprehensive Development C.I.C.; State Key Laboratory of Biological Resources and Ecological Environment of Qinling-Bashan, Hanzhong, Shaanxi, China

**Keywords:** *Atkinsoniella nigrita*, Cicadellidae, mitogenome

## Abstract

The complete mitochondrial genome (mitogenome) of the leafhopper *Atkinsoniella nigrita* Zhang & Kuoh, 1993 was determined in this study. The entire sequence was 16,011 base pairs (bp) in length. The new mitogenome consists of a typical set of genes: 13 protein-coding genes (PCGs), two ribosomal RNA (rRNA) genes, 22 transfer RNA (tRNA) genes, and one control region of 1720 bp in length. The base composition of the mitogenome was A = 41.7%, T = 38.2%, C = 10.7%, and G = 9.4%. This is the classical structure for most insect mitogenomes without any gene arrangement observed. Compared with other known mitochondrial genomes of 15 *Atkinsoniella* species, the new mitogenome with three PCGs (*ND2*, *ND5*, and *ND4L*) shared the same gene base length, start codon and stop codon, and the shortest *12S* rRNA (729 bp) and the longest *tRNA-Lys* (73 bp) within the genus *Atkinsoniella*. A phylogenetic analysis of 31 Cicadellinae and two Ledrinae concatenated sequences of 13 PCGs of their mitogenomes using Bayesian inference (BI) revealed that *A. nigrita* belongs to the genus *Atkinsoniella* with strong nodal support (BI posterior probability = 1).

## 1. Introduction

The leafhopper genus *Atkinsoniella* belongs to the subfamily Cicadellinae (Hemiptera: Auchenorrhyncha: Cicadellidae), with a distribution in Oriental and Palaearctic regions. *Atkinsoniella nigrita* is endemic to China’s fauna of Cicadellidae, and it was first reported by Zhang and Kuoh (1993). This species may be recognized by the pronotum, with a pair of big red spots, almost red forewings, and the penis bent dorsally at the apex.

There are 88 described species of *Atkinsoniella* distributed in China. To date, the complete mitogenomic sequences of 14 *Atkinsoniella* species have been published openly. Here, the complete mitogenomic sequence of *A. nigrita* was sequenced and assembled based on Illumina NovaSeq 6000 platform data. The new mitogenome unites known mitogenomes and may provide certain molecular information for the phylogeny of Cicadellinae. This could help us further understand the mitogenomic structural features of the *Atkinsoniella* species and their phylogenetic relationships.

## 2. Materials

**Figure 1.**
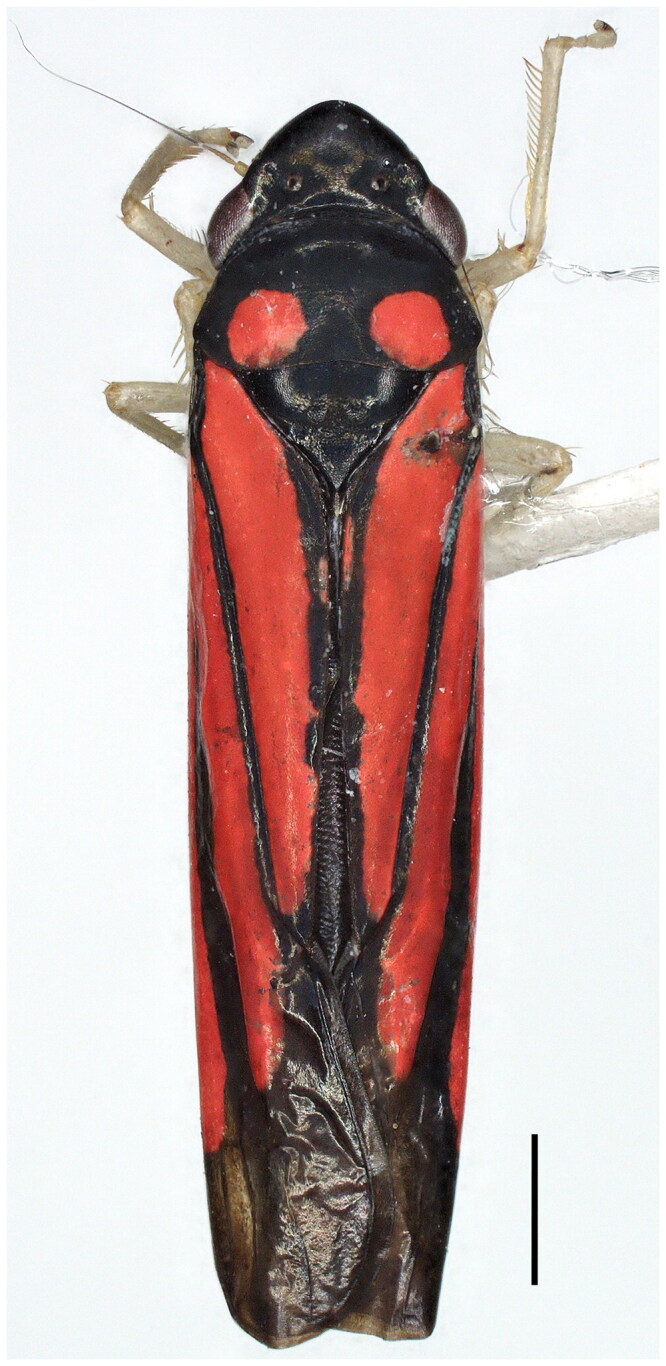
The species reference image for *A. nigrita*. The authors took the photo using a KEYENCE VHX-7000 system at the laboratory. Scale bar = 1.0 mm.

The adult specimens of *A. nigrita* in this study were collected in Lingguanxia, Baoji City, Shaanxi Province, China (106°31′22″E, 33°54′36″N), 895 m at sea level, in July 2020 (Figure 1). All fresh specimens were immediately immersed in anhydrous ethanol and stored at −20 °C in the Museum of Zoology and Botany, Shaanxi University of Technology, Hanzhong, China (SUHC) (Kai Yu, kaiyu1928@gmail.com). The sample number is 20200202.

## 3. Methods

### 3.1. Sampling and genomic DNA extraction

The specimen identification was based on the morphological characteristics, especially the male genitalia, and was referred to in the description by Zhang and Kuoh (1993). The sampled specimens were photographed (Figure 1) using a KEYENCE VHX-7000 system. The thorax muscle tissue of adult specimens was used for sample preparation, and genomic DNA was extracted using a TIANamp Genomic DNA kit (Tiangen, Beijing, China).

### 3.2. Genome sequencing and annotation

The entire mitogenome of *A. nigrita* was sequenced using the Illumina NovaSeq 6000 platform, with 50 million pieces of 150 base pairs (bp) high-throughput data sets built. Complete mitogenomes were assembled by Geneious Prime (Kearse et al. 2012) using *Atkinsoniella curvata* (OL677864) and *Atkinsoniella longiuscula* (OL677866) as references to confirm accuracy. The structure of the transfer RNA (tRNA) genes was predicted using ARWEN1.2 (Laslett and Canback 2008). rRNA genes were determined using the location of tRNAs and aligned with homologous genes of related species in GenBank. The control region was located between the *12S* rRNA and *tRNA-Ile.* The PCGs were annotated according to the open reading frame (ORF).

### 3.3. Phylogenetic analyses

In the phylogenetic analyses, 31 mitogenomes of leafhoppers representing 10 genera of the subfamilies Cicadellinae were selected as the in-group. *Ledra auditura* (MK387845) and *Ledra trigona* (MG813491) from the Ledrinae were used as the outgroup. The alignments and optimization of 13 PCGs were aligned using the MAFFT and MASCE (Vincent et al. 2011) algorithms in PhyloSuite 1.2.1 (Zhang et al. 2020) with the invertebrate mitochondrial genetic code. Then, the alignments of each gene were concatenated datasets utilizing Geneious Prime.

The best schemes for the partition and substitution models were determined in PartitionFinder v. 2.1.1 (Lanfear et al. 2012). The phylogenetic tree was inferred using MrBayes 3.26 (Ronquist and Huelsenbeck 2003). Bayesian inference (BI) analysis was performed using the following settings: Bayesian phylogenetic inference mixed with the default settings by simulating runs for 100 million generations with sampling every 1000 generations, and the convergence value was lower than 0.01 at the end of the operation.

## 4. Results

### 4.1. Mitogenome organization and nucleotide composition

**Figure 2.**
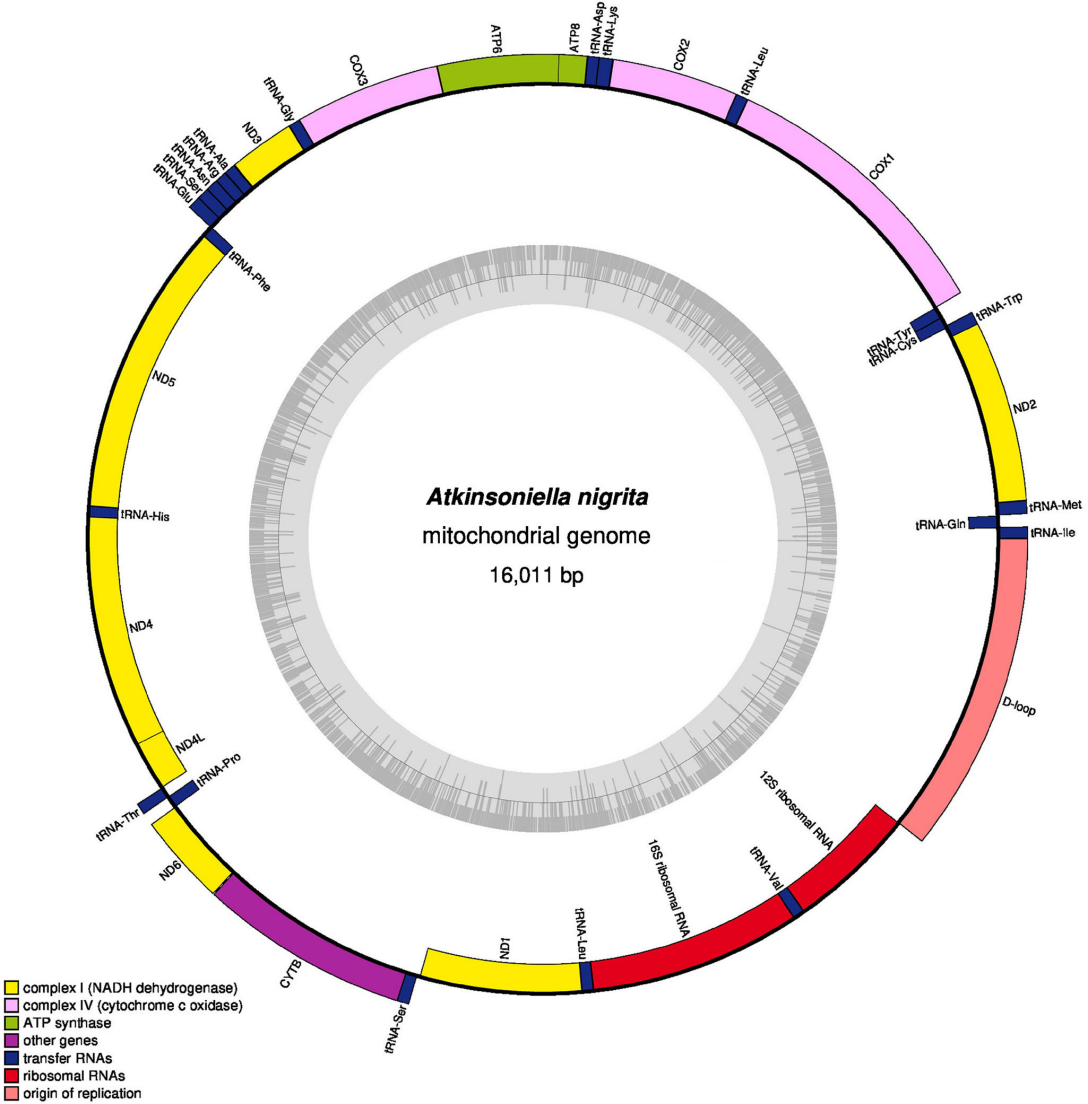
The circular map of the complete mitochondrial genome of *A. nigrita*. Different color blocks represent genes. Color blocks outside the circle indicate that the genes are on the majority strand (J-strand); those within the circle indicate that the genes are located on the minority strand (N-strand).

The length of the whole mitogenomic sequence of *A. nigrita* is 16,011 bp (Figure 2), which has been deposited in GenBank with accession number ON009029, the read coverage depth map is shown in Figure S1. This new mitogenome contained 37 typical genes (two ribosomal RNA (rRNA) genes, 22 transport RNAs (tRNAs), 13 protein-coding genes (PCGs)) and one control region (D-loop). The nucleotide composition of the new mitogenome here is biased toward A + T (A = 41.7%; T = 38.2%; C = 10.7%; G = 9.4%), and the G + C content was 20.1%.

### 4.2. Protein-coding genes and codon usage

Within the mitogenomic sequences of the genus *Atkinsoniella*, most PCGs began with a typical ATN (T/C/G/A) start codon and ended with a stop codon TAA or incomplete T. Incomplete T is a common stop codon in insects. Among the 15 mitogenomic sequences of the *Atkinsoniella* species, three PCGs are comparatively conserved. The gene base length and the start and stop codons were the same, which are *ND2* (972 bp start codon is ATT, stop codon is TAA), *ND4L* (the 282 bp start codon was ATG, the stop codon was TAA), and *ND5* (the 1675 bp start codon was TTG, the stop codon was T).

### 4.3. tRNAs and rRNAs

Among 22 tRNAs, eight tRNAs (*tRNA-Gln*, *tRNA-Cys*, *tRNa-Tyr*, *tRNA-Phe*, *tRNA-Pro*, *tRNA-His*, *tRNA-Leu2*, and *tRNA-Val*) were encoded on the N-strand, and the remaining were encoded on the J-strand. The secondary structure of tRNA genes was a typical cloverleaf structure comprising a discriminator nucleotide, acceptor stem, T ψ C arm, variable loop, anticodon arm, and DHU arm. The DHU arm was missing only the tRNA-S1 gene in the sequenced species. In contrast, the remaining were standard structures consistent with other *Atkinsoniella* species. The length of all tRNAs was found in the range of 61*–*73 bp (*tRNA-His* in 13, *tRNA-Ala* in four *Atkinsoniella* species, and *tRNA-Lys* in *A. nigrita*, respectively) when compared with the sequenced tRNA genes of *Atkinsoniella*.

The two rRNA genes contained *16S* and *12S* rRNAs between the *tRNA-Leu2* and *tRNA-Val* or between the *tRNA-Val* and control regions, respectively (Figure 2). The *16S* rRNA lengths ranged from 1188 bp (*A. warpa*) to 1217 bp (*A. xanthonota*) within the *Atkinsoniella* mitogenomes, and that of *12S* rRNAs ranged from 729 bp (*A. nigrita*) to 787 bp (*A. xanthonota*).

### 4.4. Noncoding region

The noncoding region contains two parts: gene intervals and a control region. *A. nigrita* has six gene spacers and 18 gene overlaps with the same length of 1–16 bp. Compared to its gene intervals, the control region is the longest noncoding region with 1720 bp, which plays an indispensable role. The control region of 15 Cicadellinae mitogenomes was located between 12S rRNA and tRNA-Ile and was variable in length, ranging from 744 bp to 2075 bp.

### 4.5. Phylogenetic analysis

**Figure 3.**
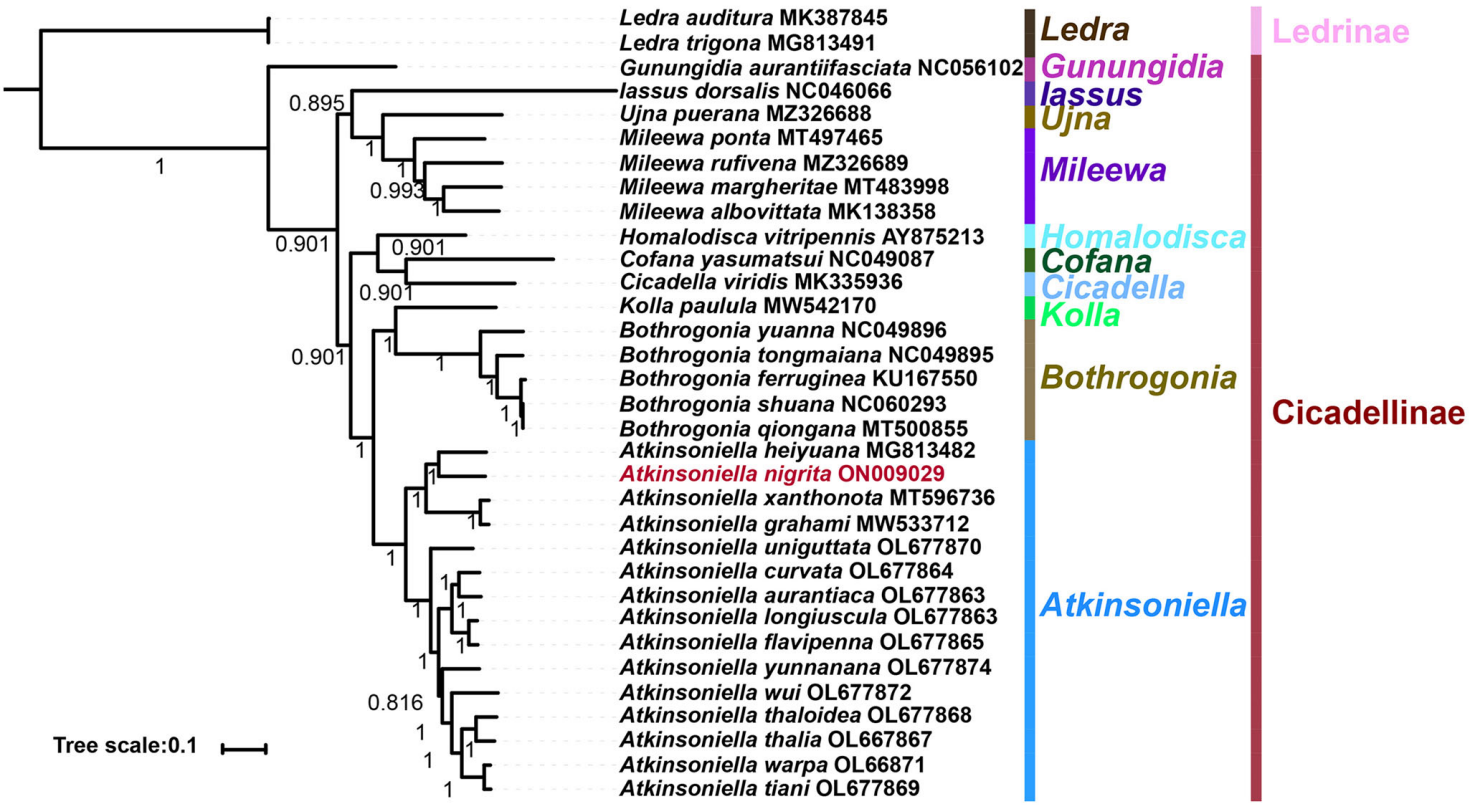
Phylogenetic tree of Cicadellidae based on 33 complete mitogenomes using Bayesian inference. The numbers at the nodes represent bootstrap values.

Bayesian inference analyses were used to reconstruct the phylogenetic relationships among the 31 species of the 10 genera of Cicadellinae and the two outgroups under the best partitioning scheme and models selected by PartitionFinder (Figure 3). The phylogenetic trees were established, and most nodes had high nodal support values. Each genus was consistently recovered as monophyletic in the obtained topology. The 10 genera relationship of this study is *Gunungidia* + ((*Iassus* + (*Ujna* + *Mileewa*)) + ((*Homalodisca* + (*Cofana* + *Cicadella*)) + ((*Kolla*+ *Bothrogonia*) + *Atkinsoniella*))). This result agrees with previous studies on Cicadellinae (Jiang et al. 2021, 2022).

## 5. Discussion and conclusions

In this study, the complete mitogenomic sequences of *A. nigrita* were sequenced, along with a comparative analysis within the 15 available mitogenome sequences of the genus *Atkinsoniella*. The length of the complete mitogenome was 16,011 bp for *A. nigrita*. Compared with other previously reported sequences of mitogenomes of the *Atkinsoniella* species, *ND2*, *ND5*, and *ND4L* are relatively conserved with the same gene base length and start and stop codons. In contrast, *A. nigrita* had the shortest *12S* rRNA and the longest *tRNA-Lys* in the base pair within *Atkinsoniella*. All the Cicadellinae mitogenomes were highly conserved in the holistic organization. Most mitogenomes were composed of 37 typically encoded genes and a control region.

A phylogenetic analysis of Cicadellinae showed that all genera are monophyletic in general. *A. nigrita* and eight other *Atkinsoniella* species were clustered into one branch of the *Atkinsoniella* with strong support (BI posterior probability = 1) (Figure 3). This is consistent with the results of previous studies (Jiang et al. 2022; Wang et al. 2022).

## Supplementary Material

Supplemental Material

## Data Availability

Mitogenome data supporting this study are openly available in GenBank at nucleotide database, https://www.ncbi.nlm.nih.gov/nuccore/ON009029, Associated BioProject http://www.ncbi.nlm.nih.gov/bioproject/PRJNA830772, BioSample accession number at https://www.ncbi.nlm.nih.gov/biosample/SAMN26806363, and Sequence Read Archive at http://www.ncbi.nlm.nih.gov/sra/SRR19660113.
