# Supplementary figures and images for: The complete mitochondrial genome of leafhopper Atkinsoniella nigrita (Hemiptera: Cicadellidae) with the shortest 12S rRNA and longest tRNA-Lys of the Atkinsoniella genus
Source: Mitochondrial DNA B Resour. 2023 Jun 29;8(6):709–13. doi: 10.1080/23802359.2023.2228932 (PMC10312024; doi:10.1080/23802359.2023.2228932)

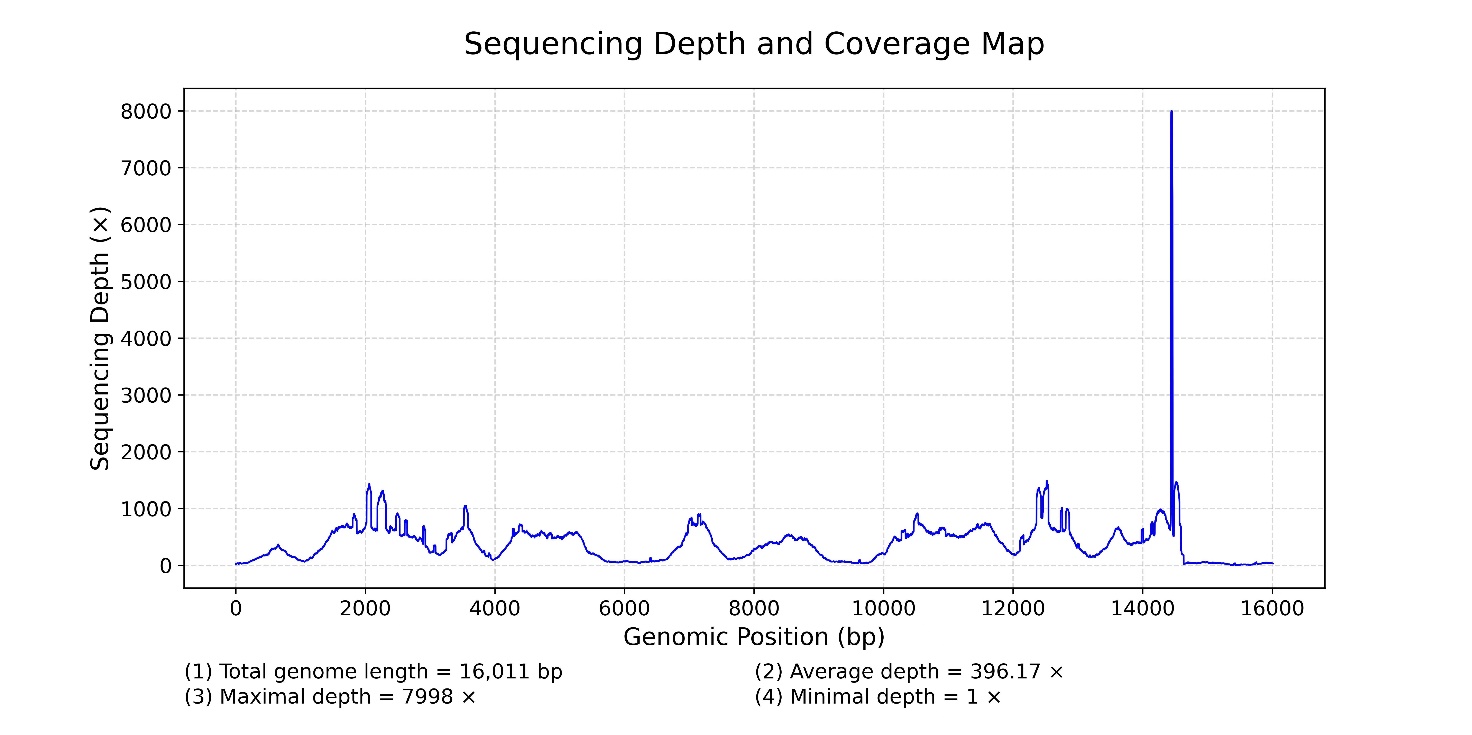


**Figure S1.** The read coverage depth map of *Atkinsoniella nigrita*.

Supplement: Supplemental Material [file TMDN_A_2228932_SM1465.docx]
